# ICARus: a pipeline to extract robust gene expression signatures from transcriptome datasets

**DOI:** 10.3389/fbinf.2025.1604418

**Published:** 2025-06-19

**Authors:** Zhaorong Li, Juan I. Fuxman Bass

**Affiliations:** ^1^ Bioinformatics Program, Boston University, Boston, MA, United States; ^2^ Department of Biology, Boston University, Boston, MA, United States; ^3^ Program in Molecular Biology, Cell Biology and Biochemistry, Boston University, Boston, MA, United States; ^4^ Biological Design Center, Boston University, Boston, MA, United States

**Keywords:** independent component analysis, transcriptomics, signatures, machine learning, robustness

## Abstract

Gene signature extraction from transcriptomics datasets has been instrumental to identify sets of co-regulated genes, identify associations with prognosis, and for biomarker discovery. Independent component analysis (ICA) is a powerful tool to extract such signatures to uncover hidden patterns in complex data and identify coherent gene sets. The ICARus package offers a robust pipeline to perform ICA on transcriptome datasets. While other packages perform ICA using one value of the main parameter (i.e., the number of signatures), ICARus identifies a range of near-optimal parameter values, iterates through these values, and assesses the robustness and reproducibility of the signature components identified. To test the performance of ICARus, we analyzed transcriptome datasets obtained from COVID-19 patients with different outcomes and from lung adenocarcinoma. We identified several reproducible gene expression signatures significantly associated with prognosis, temporal patterns, and cell type composition. The GSEA of these signatures matched findings from previous clinical studies and revealed potentially new biological mechanisms. ICARus with a vignette is available on Github https://github.com/Zha0rong/ICArus.

## Introduction

Transcriptomic data plays a crucial role in understanding the variation in gene expression patterns across diverse biological conditions and phenotypes. Common approaches to analyze such data involve conducting a differential expression and gene expression pattern analyses, which evaluate changes in expression across groups (Conesa et al., 2016). However, challenges arise when analyzing large transcriptomic datasets from sources like Genotype-Tissue Expression (GTEx) (Lonsdale et al., 2013) and the Cancer Genome Atlas (TCGA), since these data can often be classified according to multiple known (e.g., tissue, sex, age, tumor type, tumor stage, *etc.*) as well as unknown variables. This complicates identifying the contribution of the different variables to the differences in expression observed across samples. To address these issues and enable the analysis of such large datasets, unsupervised algorithms like principal component analysis (PCA), Weighted Gene Co-Expression Network Analysis (WGCNA) (Langfelder and Horvath, 2008), non-negative matrix factorization (NMF) (Jia et al., 2015), and independent component analyses (ICA) (Anglada-Girotto et al., 2022) have been developed. Unlike methods that compare gene expression between groups, these unsupervised algorithms identify gene expression modules or signatures associated with the phenotype labels of the samples.

ICA has been widely used to identify gene expression signatures in large transcriptomic datasets, including cancer, development, and exposure to treatments (Biton et al., 2014). ICA separates a multivariate signal, in this case gene expression, into additive subcomponents or signatures which are positive and negative contributions of each gene in the dataset. One key parameter in ICA is determining the optimal number of signatures to extract in a dataset as there is no ground truth for the actual number of independent contributing variables. Most pipelines, such as RobustICA (Anglada-Girotto et al., 2022) and BIODICA (Kairov et al., 2012) select this optimal parameter based on the number of components needed in PCA to explain a percentage of variance in the dataset. These studies often increase robustness by iterating the analysis using the same parameter; however, signatures often vary widely across parameter values. This can lead to the identification of low-confidence, non-reproducible signatures.

**FIGURE 1:**
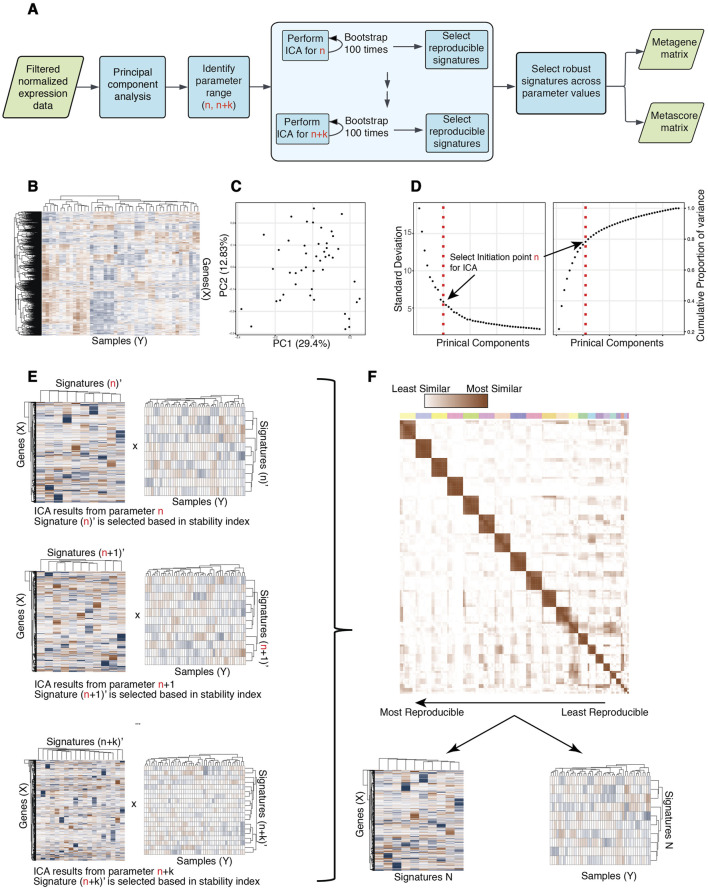
Overview of ICARus pipeline. **(A)** Pipeline diagram overview of ICARus. **(B)** The input for ICARus is a (Genes x Samples) normalized gene expression matrix where the rows are gene symbols (IDs) and columns are samples. **(C)** PCA plot of samples based on normalized gene expression. **(D)** Left = Standard deviation of each principal component sorted by principal component rank order. Right = Cumulative proportion of variance explained across principal components. The elbow and knee-points in these plots are used to identify the initiation point n for ICA. **(E)** For each parameter between n and n + k (k is defined by user) ICA is performed 100 times, and Icasso quality index is used to assess the robustness of independent components. **(F)** The independent components that pass the user defined robustness threshold for each tested parameter value are clustered. The sizes of clusters indicate the reproducibility of signatures across different parameter values. Signatures that pass the user defined reproducibility scores are output as genes x signatures and signatures x samples matrices.

Here, we introduce the R package ICARus (Figure 1A), designed to streamline the application of ICA and extraction of high-confidence expression signatures that are robust across iterations and reproducible across parameter values. ICARus leverages the proportion of variance explained obtained from PCA to provide a range of near-optimal parameters for the ICA algorithm. Subsequently, for each parameter the ICA algorithm is applied, and the results are clustered and evaluated using the stability index proposed by Icasso to identify robust signatures for each parameter (Himberg and Hyvarinen, 2003). ICARus then clusters the robust signatures obtained to identify reproducible signatures across parameters. Finally, the gene expression signatures with the highest reproducibility scores are combined into meta-signatures and subjected to further analysis through Gene Set Enrichment Analysis (GSEA) or Fisher’s Exact test to functionally interpret the signatures identified.

## Materials and methods

### Input data format for ICARus

The input data for ICARus is a normalized transcriptome dataset in matrix format, with rows being gene names and columns being samples (Figure 1B). Normalization methods such as Counts-per-Million (CPM) (Chen et al., 2025) and Ratio of median (Anders and Huber, 2010) are recommended. Different normalization method strategies will introduce differences in the final results of ICARus; however, most of the signatures are reproducible in the results obtained from different methods (Supplementary Figure S1).

Prefiltering of sparsely expressed genes in the input data is recommended as these genes introduce noise in the analysis, but since the filtering strategy varies between different datasets, it is not included in the pipeline.

### Estimating the set of near-optimal parameters for the ICA algorithm

To estimate the set of near-optimal parameters, ICARus first performs PCA for the input dataset (Figure 1C). Prior work has used an optimal parameter N as the number of top principal components that collectively account for 99% of the variance observed in the dataset (Sastry et al., 2019). ICARus, also relies on the variance explained by PCA, but identifies the range of near-optimal values for n. After performing PCA, users can select whether to use: 1) the ranked distribution of standard deviations of each principal component, or 2) the cumulative proportion of variance explained by a certain number of principal components to determine the lower bound for the parameter set. In the first option, the standard deviation of each principal component is plotted against the ranked order of the principal components which takes the form of an elbow plot; whereas in the second option, the cumulative proportion of variance explained against the order of principal components takes the form of a knee plot. The elbow-point in the first plot and the knee-point in the second plot indicates the top n principal components that explain a large fraction of the variance in the data (Figure 1D), i.e., including more principal components does not lead to a marked increase in variance explained.

To pinpoint this critical elbow/knee point, the Kneedle Algorithm (Satopaa et al., 2011) is used, and this identified point is designated as the minimum number n for the near-optimal parameter set for subsequent ICA analysis. The Kneedle algorithm was implemented in an R package^1^. This set of parameters is then selected as every integer (n, n + k) where k can be user defined and is set as default to be 10.

### Generating reproducible gene signatures

Following the identification of the near-optimal parameter set, ICARus initiates the generation of reproducible gene signatures employing two sequential strategies: intra-parameter iterations and inter-parameter iterations. For the intra-parameter iterations, ICARus conducts the ICA algorithm 100 times for each n value. Subsequently, the resulting signatures undergo sign correction suggested by a previous study (Anglada-Girotto et al., 2022) and hierarchical clustering to identify sets of robust signatures for each specific n. Within each cluster, the medoid is extracted and employed as the representative signature, while the stability of the signature cluster is assessed using the stability index proposed by Icasso (Figure 1E) (Himberg and Hyvarinen, 2003). To calculate the stability index, the similarities between signatures from different runs are calculated using the absolute value of the Pearson correlation coefficient $\sigma_{i,j}$ . Then the stability index of given cluster M is calculated using the following function in Equation 1:

$$\frac{1}{{\left|C_{M}\right|}^{2}}\sum_{i,j\in C_{M}}\sigma_{i,j}-\frac{1}{\left|C_{M}\|C_{-M}\right|}\sum_{i\in C_{M}}\sum_{j\in C_{-M}}\sigma_{i,j}$$ (1)

where $\left|C_{M}\right|$ and $\left|C_{-M}\right|$ are the size of cluster M and the number of signatures not in cluster M (Himberg and Hyvarinen, 2003). The stability index calculated by this function ranges from 0 to 1, from least to most stable. The signatures with stability indices >0.75 are evaluated for reproducibility across values of n (Figure 1F). These robust signatures are subjected to hierarchical clustering. A signature obtained with one value of the parameter is considered reproducible if it clusters together with signatures obtained across multiple other n values within the near-optimal set. The user can specify whether to only keep the reproducible signatures originated from the starting point n, or to also keep the reproducible signatures originated from a higher parameter within the near-optimal set, as long as they can be reproduced in more than half of the remaining tested parameters.

ICARus outputs the number of near-optimal values that contribute a signature to the cluster and the average distance between these signatures for each cluster. These values can then be used to select reproducible signatures across many parameter values (Figure 1F).

### Output signatures and downstream analysis of the gene signatures

The reproducible signatures extracted by ICARus consist of two parts: 1) a matrix of genes by signatures, where each value indicates the contribution of the gene to the signature (the distribution in scores of the signatures follows the normal distribution, with the mean of 0); and 2) a matrix of signatures by samples where each value indicates the contribution of the signature to the expression profile of the sample (Figure 1F). The gene scores of a particular signature can be used to perform Gene Set Enrichment Analysis (Subramanian et al., 2005) to identify pathways or gene sets associated with the signature for further biological interpretation. The signatures scores across samples can be used to associate signature values with sample phenotypes or temporal patterns.

### Implementation

Steps that are described above were implemented in R with parallel backend computation as package *ICARus* and provided as pseudocode in Supplementary Material S1. The package and a vignette is available on Github https://github.com/Zha0rong/ICArus.

### Test datasets

#### Peripheral leukocyte samples from COVID-19 patients

**FIGURE 2:**
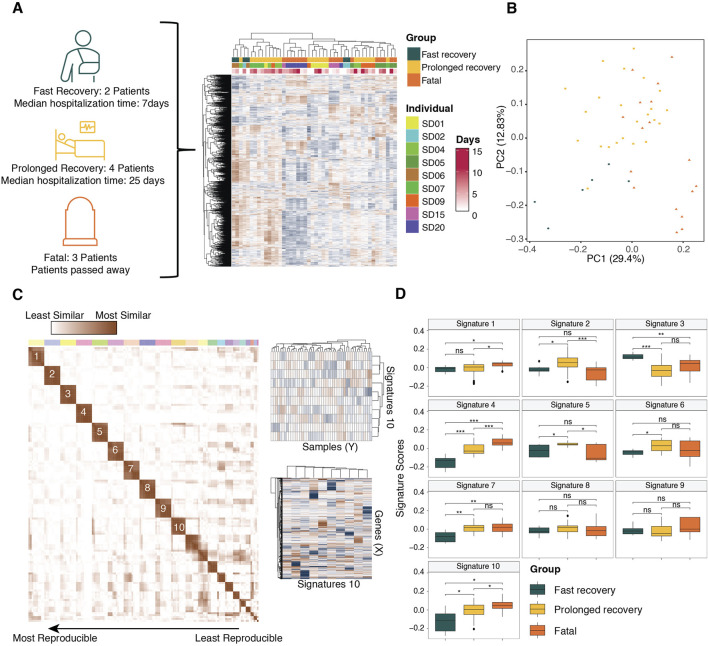
Application of ICARus to a COVID-19 transcriptomic dataset. **(A)** The test dataset consists of 46 samples of blood-derived leukocytes obtained from COVID-19 patients with different clinical outcomes at different time points during infection. After filtering genes with no expression in more than half of the dataset, 17,557 genes were kept for downstream analysis. **(B)** The PCA plot illustrates the separation of samples from different clinical outcomes. **(C)** The initiation parameter identified by ICARus for this dataset was 10 (n), and ICARus determined robust signatures using parameter values 10–19. Signatures across parameter values were clustered and only the signatures that were reproducible across more than 5 values were considered for downstream analysis. ICARus identified 10 robust and reproducible gene expression signatures from the test dataset. **(D)** The box plots showed the signature score distributions in different clinical outcomes. Statistical significance determined by Wilcoxon-ranked sum test.  *p < 0.05,  **p < 0.01,  ***p < 0.005.

To illustrate the efficacy of ICARus in identifying relevant signatures, we applied it to a publicly available RNA-Seq dataset featuring 46 peripheral blood leukocyte samples collected from 11 COVID-19 patients infected with SARS-CoV-2, with varying clinical outcomes (fast recovery, prolonged recovery, and fatal) at different time points (Figure 2A) (Lam et al., 2023). Fast recovery patients had a median hospitalization time of 7 days, prolonged recovery patients had a median hospitalization time of 25 days, and fatal patients were patients that passed away due to complications of the infection. The count matrix was downloaded from the GEO repository (GSE221066), which included 26,475 genes and 55 samples. To prevent genes with sparse expression introducing noise in the analysis, only genes with non-zero expression in at least one-fourth of the samples were included in the analysis. This strategy filtered out 8,918 genes and retained 17,557 genes for the analysis. The count matrix was normalized using the Counts-Per-Million method (Chen et al., 2025).

#### Primary tumor samples of lung adenocarcinoma (LUAD)

To test the performance of ICARus on a large RNA-seq dataset with complex clinical phenotypes, we processed 539 lung adenocarcinoma primary tumor RNA-seq samples from TCGA database (Cancer Genome Atlas Research Network, 2014), which were downloaded through the TCGA-biolinks portal (Colaprico et al., 2016). The count matrix included 19,938 protein coding genes and 539 samples. To filter out genes with sparse and low expression in the dataset, only genes with non-zero expression in at least one-fourth of the samples were included in the analysis. This strategy filtered out 1,417 genes and retained 18,091 genes and 539 samples for the analysis. The count matrix was normalized using the Counts-Per-Million method (Chen et al., 2025).

## Results

### Identification of reproducible signatures in a COVID-19 expression dataset

To identify signatures associated with COVID-19 outcomes, we used a dataset of 46 samples derived from 11 patients with different outcomes (fast recovery, prolonged recovery, and fatal) at different time points (Figure 2A) (Lam et al., 2023). First, we determined the near-optimal parameter set in the COVID-19 expression dataset. We then selected the critical elbow-point in the PCA option provided by ICARus. This corresponded to 10 principal components; therefore, the nominated range for the ICA parameter was 10–19 independent components. We used 100 iterations for each of these parameter values, then identified the medoid signature, followed by clustering of signatures across the parameter values. This resulted in 10 signatures that were reproducible across more than half of the tested parameter values (Figures 2B,C). By comparing the signature scores between samples from patients with different clinical outcomes, we identified two signatures (signatures 4 and 10) that monotonically increase with outcome severity (Figure 2D). Next, we aimed to determine the biological processes associated with these signatures.

#### Signature 4 is associated with poor prognosis and fatal outcomes

**FIGURE 3:**
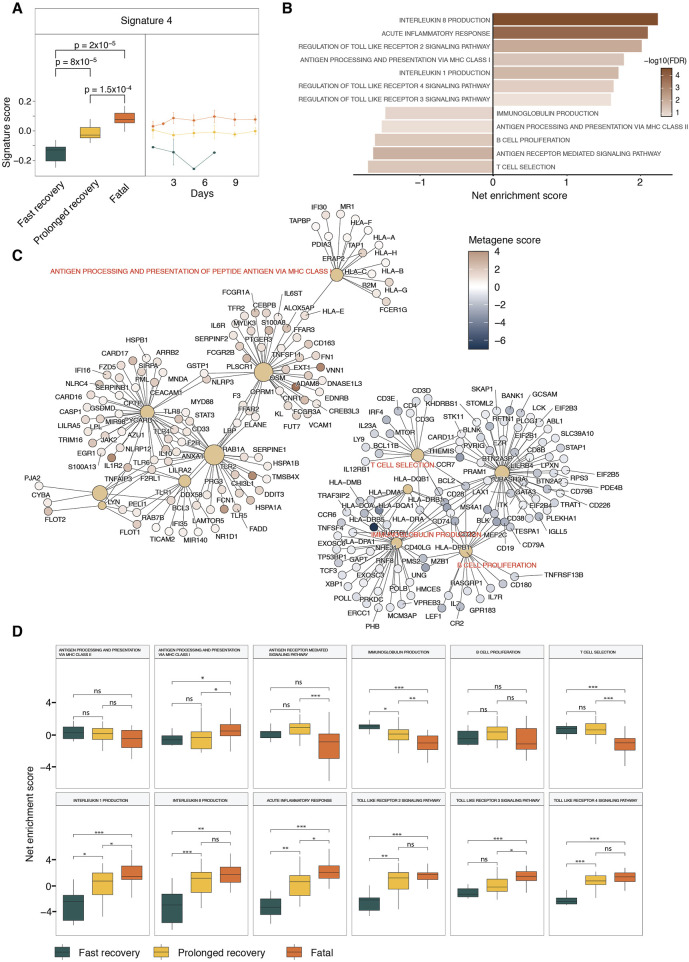
GSEA of clinical outcome-associated Signature 4. **(A)** The box plots show the signature 4 score distributions in different clinical outcomes. The line plot shows the temporal pattern of Signature 4 for different patient outcomes. Statistical significance determined by Wilcoxon-ranked sum test. **(B)** Bar graph displays the top enriched and depleted pathways from GSEA analysis results of Signature 4. Net enrichment scores are shown. **(C)** Network representation of the top enriched pathways and the driver genes associated with the enrichment results. **(D)** ssGSEA results of the top enriched pathways from GSEA analysis results of Signature 4.

Signature 4 exhibited a significant correlation with patient outcomes, with samples from fatal outcome patients having the highest scores and those from fast-recovery patients having the lowest scores (Figure 3A). By plotting signature scores across time points and clinical outcomes, we observed that signature 4 scores were higher in samples from fatal outcome patients, and lower in fast-recovery patients at every time point (Figure 3A). This observation is important as it rules out the possibilities of association driven by the bias at one or more time points and suggests that the biological functions associated with signature 4 can be used to differentiate fast recovery patients at any time point.

The GSEA analysis of signature 4 revealed a depletion of T and B cell activation and MHC class II antigen processing and presentation, and an enrichment of inflammation pathways and MHC Class I antigen presentation (Figure 3B). To identify which genes were driving the observed enrichments, a net plot was generated (Figure 3C). In this plot, large brown nodes represent enrichment terms, while smaller red or blue nodes represent individual genes colored according to their scores in signature 4. Edges were drawn between nodes when a gene belonged to the core enrichment set of a given term. In the network graph, elevated scores of immune genes, such as IL1R2, MYD88, NRLP3, CASP1, TLR4, were shown to drive the enrichment of interleukin 1 and interleukin 8 producing signaling pathways (Figure 3C). This is consistent with previous studies linking the elevated expression of IL-1 and IL-8 with poor prognosis (Li et al., 2021; Cavalli et al., 2021). Further clinical studies also showed that the blocking of IL-1 in COVID-19 patients led to better prognosis (Cavalli et al., 2020). The network also showed that toll-like receptor genes such as TLR1, TLR2 and TLR4, which had elevated metagene scores, were driving the enrichment of toll-like receptor signaling pathways (Figure 3C). The TLR2 signaling pathway, elevated in signature 4, can also be associated with poor prognosis, consistent with several clinical studies showing elevated TLR2 expression was associated with poor prognosis in COVID-19 infection (Taniguchi-Ponciano et al., 2021; Xu Q. et al., 2022). Signature 4 has also a negative association with MHC-Class II antigen presenting pathways, which is driven by the suppression of MHC-Class II such as HLA-DMA, HLA-DMB and HLA-DRA (Figure 3C), suggesting a negative association with poor prognosis. This is consistent with previous studies showing that monocytes in COVID-19 patients have lower levels of MHC class II proteins (Xu Q. et al., 2022; Laing et al., 2020). These results were confirmed using ssGSEA (Reich et al., 2006) that calculate net enrichment scores of individual pathways in each sample (Figure 3D).

#### ICARus identified signature 10 as associated with a temporal phenotype

Signature 10 not only displayed an association with clinical outcomes (lowest in fast recovery, highest in fatal), but also showed a temporal phenotype (Supplementary Figure S2A). Prolonged recovery patients and fatal outcome patients had similar signature 10 scores in the beginning time point, but fatal outcome patients had consistent higher signature 10 scores in the later time points (Supplementary Figure S2A). GSEA analysis of signature 10 revealed positive associations with regulation of neutrophils chemotaxis/mediated immunity, actin filaments assembly/organization and extracellular matrix (Supplementary Figure S2B). A net plot was generated for the genes and the enriched terms to visualize the genes that drive the enrichment of given pathways (Supplementary Figure S2C). For example, matrix metalloproteinase genes such as MMP2 and MMP8 drive the enrichment of extracellular matrix disassembly and galectin genes such as LGALS1, LGALS3 and LGALS9 drive the enrichment of neutrophil mediated immune pathways. Previous studies (Schulte-Schrepping et al., 2020) have shown that elevated neutrophil counts are associated with a poor prognosis in COVID-19 patients, with clinical publications attributing the poor prognosis to the formation of neutrophil extracellular traps (NETs) (Zuo et al., 2021). NETs, composed of cell-free DNA, histones, and cytosolic proteins released by neutrophils, require the rearrangement of the actin cytoskeleton for their formation (Sprenkeler et al., 2022). NETs have been implicated in thrombosis and tissue damage (Papayannopoulos, 2018; Zuo et al., 2020), contributing to the poor prognosis of COVID-19 patients.

GSEA analysis results also revealed a negative association between signature 10 and regulation of T cell activation and T cell mediated immunity, driven by suppression of killer cell lectin-like receptors such as KLRC2/3/4, KLRD1 and KLRK1 (Supplementary Figure S2C). Previous studies have also shown decreasing T cell counts in COVID-19 patients with severe symptoms compared to the ones with non-severe symptoms (Liu et al., 2020). Further, another study reported an elevated number of neutrophils and decreasing number of T cells in COVID-19 patients with severe symptoms compared with COVID-19 patients with mild symptoms (Xu J. et al., 2022).

## Identification of reproducible signatures in a TCGA-LUAD expression dataset

**FIGURE 4:**
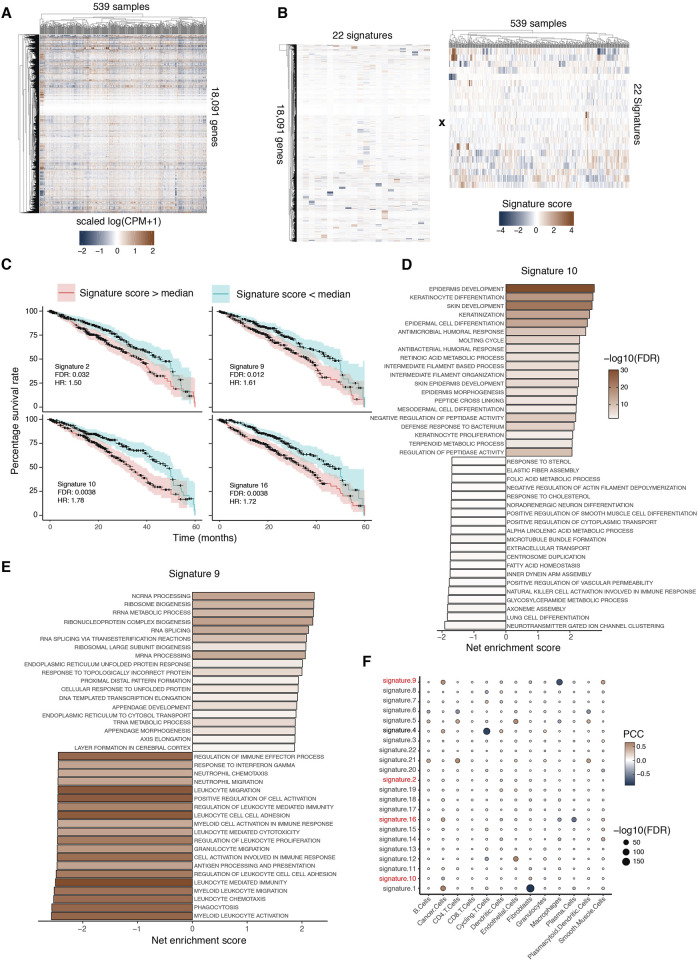
ICARUS extracts prognosis-related signatures from TCGA-LUAD database. **(A)** 539 primary tumor RNA-Seq samples were downloaded from the TCGA database, and 18,091 genes were kept in the analysis. **(B)** ICARUS identified 22 reproducible signatures. **(C)** Kaplan Meier plots of 4 signatures significantly associated with adversary prognosis. The adjusted p-values obtained from the Cox-proportional hazard ratio test, covariate factors such as gender, age, tissue of origin were regressed out using likelihood ratio test. HR = hazard ratio. **(D and E)** The bar graph displays the top enriched and depleted pathways from GSEA results of Signature 10 **(D)** and Signature 9 **(E)**. Net enrichment scores are shown. **(F)** Dot plot of Pearson correlation coefficient (PCC) and adjusted p-value of correlation tests between signature scores and cell type proportion. The color of the dot showed correlation coefficients and the size of the dots showed −log_10_ (adjusted p-value).

To demonstrate the application of ICARus on another larger dataset, we selected the TCGA-LUAD lung adenocarcinoma expression dataset (Figure 4A) (Cancer Genome Atlas Research Network, 2014). To identify the near-optimal parameter set, we selected the critical elbow-point in the PCA option provided by ICARus. This corresponded to 48 principal components, and therefore, the nominated range for the ICA parameter was 48–57 independent components. We performed 100 iterations for each of these parameter values, identified the medoid signature, then clustered signatures across the parameter values. This resulted in 22 signatures that were reproducible across more than half of the parameter values tested (Figure 4B).

## Identification of gene signatures associated with disequilibrium of cell type proportion and adverse prognosis

To identify signatures associated with adverse prognosis, samples were stratified by the median score of each gene signature into two groups: samples with higher given gene signature scores and samples with lower given gene signature scores. The Cox proportional hazards model was used to perform the survival analysis and the likelihood ratio test was used to regress out co-variables such as age of diagnosis, gender, location of tumor origin, and cell type proportion. Four signatures were significantly associated with adverse prognosis (Figure 4C). We performed GSEA for signatures 9 and 10 to determine the pathways associated with adverse prognosis. GSEA of signature 10 showed an enrichment of keratinization related processes and depletion of metabolic, immune-related, and cell division related pathways (Figure 4D). Previous studies have shown that keratin gene expression activates the epithelial-mesenchymal transition in tumor cells and leads to poor prognosis (Li et al., 2024). GSEA of signature 9 showed an enrichment of appendage development pathways and depletion of macrophages and immune related pathways (Figure 4E). Previous studies have shown that the enrichment of appendage development pathways was associated with poor prognosis (Yu et al., 2024).

To determine whether signature 9 is indeed associated with a depletion of macrophages in the corresponding samples, we used BayesPrism (Chu et al., 2022) to deconvolve the 539 bulk RNA-Seq samples and predict the proportion of each cell type in the tumor microenvironment of each sample. Then, we performed correlation tests between the proportion of each cell type and score of each signature. The results were visualized using the dot plot where the rows are signatures and the columns are cell type proportions, the sizes of the dot are the negative log10 transformed FDR adjusted p-values of the correlation tests and the colors of the dots are the correlation coefficients (Figure 4F). We found several signatures associated with cell type proportions. In particular, signature 9 was significantly associated with a low proportion of macrophages, consistent with our GSEA results.

## Discussion

We developed ICARus, an R package designed to assist researchers in identifying robust and reproducible gene signatures using ICA across multiple parameter values. This pipeline is highly versatile enabling users to select analysis parameters and stringency. First, ICARus enables the user to manually or automatically select near-optimal parameter sets using elbow or knee points of PCA results. Next, ICARus allows users to select the reproducibility criteria. Although our analyses focused on signatures identified in more than half of all parameters tested, the pipeline can also output signatures present in more than half of parameters from their first instance. This allows the identification of signatures specific to higher parameter values.

To show that the gene expression signatures extracted by ICARus are meaningful, we tested the package on two RNA-Seq datasets. The first dataset consisted of leukocytes samples which were obtained from COVID-19 patients with different clinical outcomes, and the second dataset consisted of primary tumor samples obtained from lung adenocarcinoma patients. The analysis of COVID-19 patient samples showed that ICARus identified biologically meaningful signatures that were associated with patient prognosis and the pathways that drive these associations.

Analysis of the primary tumor samples showed that ICARus identified gene signatures associated with prognosis and cell type proportion in the tumor microenvironment. The reproducible signatures identified by ICARus were associated with clinical phenotypes and temporal patterns consistent with previous studies. Furthermore, the network analyses of the signatures domonstrated that the signatures will provide biologically meaningful genes driving the enrichment of relevant biological functions. These genes can be used as input for some of the recently published algorithms that employ deep learning algorithms to study gene interactions networks and drug response (Zhao et al., 2024; Zhao et al., 2025; Zhao et al., 2022). In principle, ICARus can also be used to extract signatures from single cell RNA-seq datasets; however, the method may need adaptation to account for noise and missing values.

In summary, ICARus has demonstrated the ability to produce biologically meaningful and reproducible signatures which can be extended to other expression datasets.

## Data Availability

The original contributions presented in the study are included in the article/Supplementary Material, further inquiries can be directed to the corresponding author.

## Footnotes

1
https://github.com/etam4260/kneedle
