# Supplementary figures and images for: ICARus: a pipeline to extract robust gene expression signatures from transcriptome datasets
Source: Front Bioinform. 2025 Jun 19;5:1604418. doi: 10.3389/fbinf.2025.1604418 (PMC12222331; doi:10.3389/fbinf.2025.1604418)

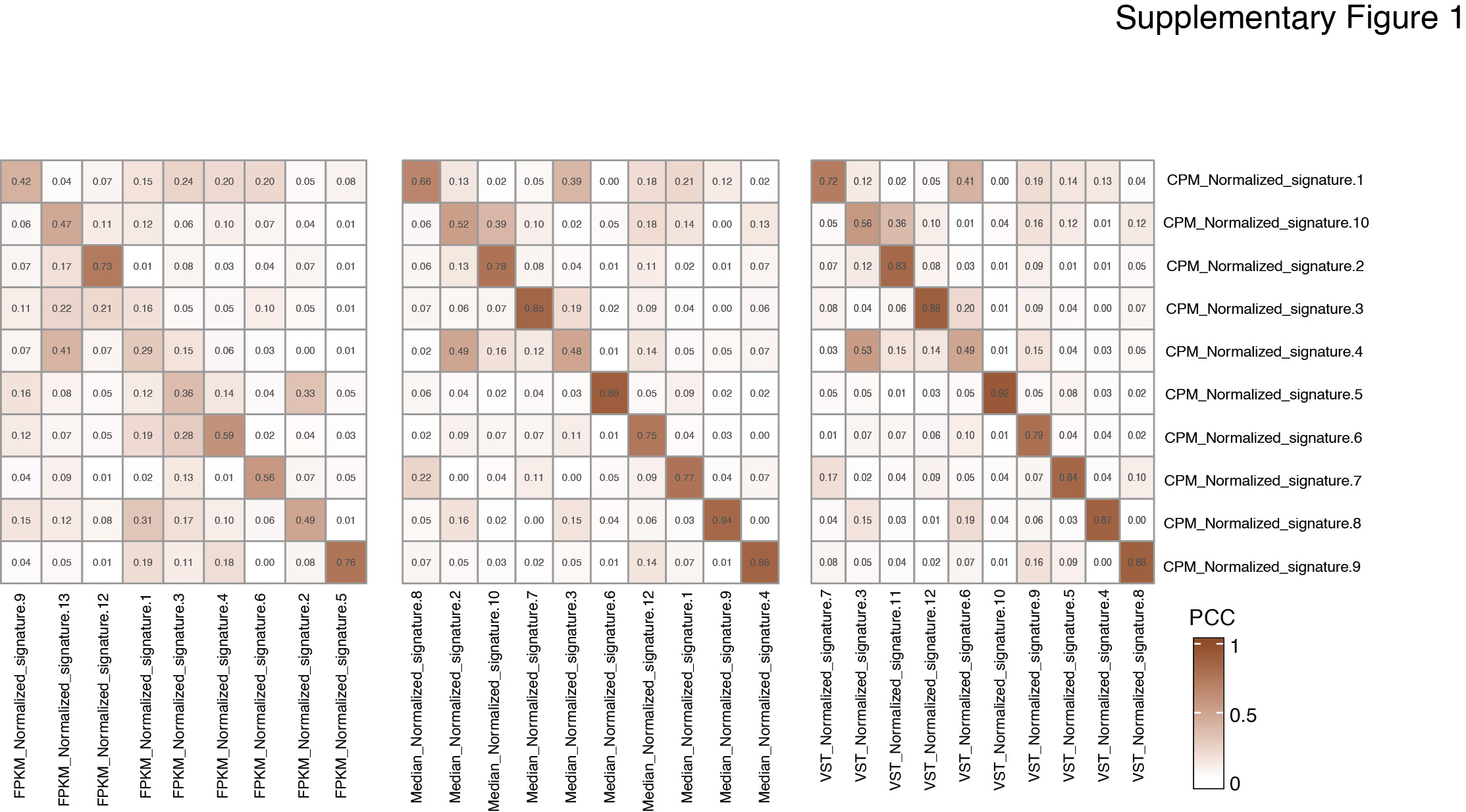

Supplement: Supplementary file 1 [file Image1.JPEG]

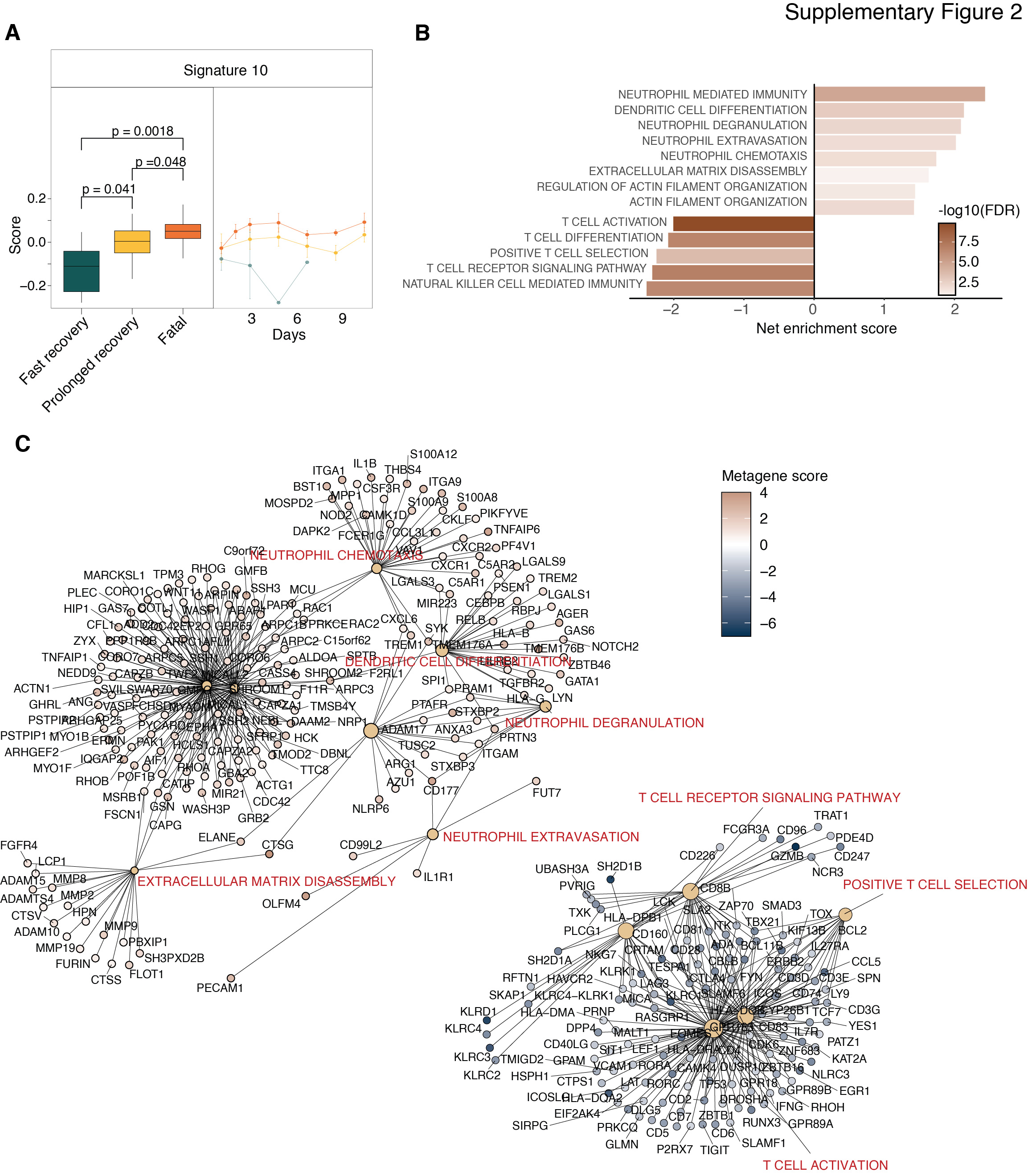

Supplement: Supplementary file 2 [file Image2.JPEG]
