# Supplementary material for: ICARus: a pipeline to extract robust gene expression signatures from transcriptome datasets
Source: Front Bioinform. 2025 Jun 19;5:1604418. doi: 10.3389/fbinf.2025.1604418 (PMC12222331; doi:10.3389/fbinf.2025.1604418)
Supplement: Supplementary file 3 [file DataSheet1.docx]

**Pseudo-code of ICARus**

Input: a scaled normalized count matrix from RNA-Seq data M, rows as genes and columns as samples (potentially cells, ICARUS can be run on single cell data).

1. Determine the near optimal number of parameters ***N***:
2. Run principal component analysis on count matrix M
3. Calculate the standard deviation and proportional variance explained by each principal component
4. Use Kneedle algorithm [11] to find the optimal parameter ***N*** based on the standard deviation (finding the elbow point, **figure 1D**) or on the proportional variance explained (finding the knee point, **figure 1D**)
5. for every integer ***n*** from ***N*** to ***N+K*** (K>0, the default value is 10):
6. Run independent component analysis 100 times using parameter ***n***, yielding ***100*n*** signatures
7. Calculate the pearson's correlation coefficients between every pair of signatures in ***100*n*** signatures
8. Cluster the signatures based on the absolute value of pearson’s correlation coefficients calculated in step (**b**) using hierarchical clustering, yielding ***n*** clusters
9. Use the function $S = \frac{1}{\left| C_{M} \right|^{2}}\sum_{i, j \in C_{M}} \sigma_{i,j}-\frac{1}{|C_{M}||C_{-M}|}\sum_{i \in C_{M}} \sum_{j \in C_{M}} \sigma_{i,j}$ to calculate the signature stability ***S*** (ranging from 0 to 1) for each cluster, where $\left| C_{M} \right|$ and $\left| C_{-M} \right|$ are the size of cluster M and the number of signatures not in cluster M, $\sigma_{i,j}$ is the correlation coefficient between signature *i* and signature *j*.
10. Remove clusters that have ***S*** smaller than 0.75, and have size does not equal 100.
11. Find the medoids of the clusters that pass the filtering in the previous step, yielding ***n’*** signatures that are reproducible in 100 runs using parameter ***n*** (***n’*** <= ***n***)
12. Calculate the correlation coefficients between every pair of signatures in signatures yielded in step 2.
13. Cluster the signatures based on the absolute value of pearson’s correlation coefficients calculated in step 3 using hierarchical clustering.
14. for every signature in ***n’*** from the results of running ICA using parameter ***N***:
    1. Find out in which cluster the signature is
    2. If the size of the given cluster is larger than k/2, that means the signature is reproducible in more than half of the runs with different parameters
    3. Keep the ***n’’*** signatures that belong to clusters with size larger than k/2. (***n’’*** >= ***n’***)
15. Report the signatures from step 5.
